# Human disturbance is the major driver of vegetation changes in the Caatinga dry forest region

**DOI:** 10.1038/s41598-023-45571-9

**Published:** 2023-10-27

**Authors:** Helder F. P. Araujo, Nathália F. Canassa, Célia C. C. Machado, Marcelo Tabarelli

**Affiliations:** 1 Present Address: Department of Biosciences, Federal University of Paraíba, Areia, Paraíba 58397-000 Brazil; 2 Postgraduate Program of Biological Sciences-Zoology, Federal University of Paraíba, João Pessoa, Paraíba Brazil; 3 Department of Biosciences, Federal University of Paraíba, Areia, PB CEP: 58397-000 Brazil; 4 Center of Applied Biological and Social Sciences, State University of Paraíba, João Pessoa, Paraíba Brazil; 5 Department of Botany, Federal University of Pernambuco, Recife, Pernambuco Brazil

**Keywords:** Environmental impact, Biogeography, Climate-change impacts

## Abstract

Drastic changes in vegetation structure caused by exceeding ecological thresholds have fueled the interest in tropical forest responses to climate and land-use changes. Here, we examine the potential successional trajectories experienced by the largest dry tropical forest region in South America, driven by climate conditions and human disturbance. We built potential distribution models for vertebrate taxa associated with forest or shrub habitats to estimate natural vegetation cover. Distribution patterns were compared to current vegetation across the entire region to identify distinct forest degradation levels. Our results indicate the region has climatic and soil conditions suitable for more forest cover than is currently found, even in some areas with limited precipitation. However, 11.04% of natural cover persists across such an immense region, with only 4.34% consisting of forest cover. Forest degradation is characterized by the dramatic expansion of shrubland (390%), farming, and non-vegetation cover due to changes in land-use, rather than climatic conditions. Although different climate conditions have been the principal drivers for natural forest distribution in the region, the forest seems unable to resist the consequences of land-use changes, particularly in lower precipitation areas. Therefore, land-use change has exceeded the ecological thresholds for the persistence of forests, while climate change may exacerbate vegetation-type transitions.

## Introduction

Climate and land-use change are expected to drastically alter patterns of natural vegetation cover, particularly in the tropics^1–3^. This emerging trend is not just related to the replacement of natural vegetation by other types of anthropogenic land use (e.g., agriculture, pasture, urban areas), but is also related to drastic changes in vegetation structure and functionality as ecological thresholds (i.e., tipping points) are exceeded^4–6^. In general, projections indicate dry forests may be replaced by deserts (bare soils) in response to reduced precipitation and increased human disturbance, such as habitat loss and fragmentation, logging, harvesting of forest products, fire, and other chronic disturbances^7^. Such trajectories are expected to change the ability of natural vegetation to deliver ecosystem services, with impacts on both regional and global sustainability. Accordingly, there has been increasing interest in the drivers of changes in vegetation cover and their local- and global-scale effects, including the relative contribution of both climate and local human pressure on native vegetation.

Drylands have been expanding across the globe since the 1960s, and are projected to expand further throughout the twenty-first century under future climate changes^8–11^. This transition towards treeless vegetation states is argued to emerge as a result of reductions in precipitation^12,13^. However, such projections are based on the associations between present-day tree cover distribution and precipitation data, without the consideration of anthropogenic pressures, which may lead to a misinterpretation of the importance of climate for the distribution of natural semiarid vegetation, i.e. precipitation being assumed to be the main driver determining the distribution of natural vegetation in dry forests, when long-term anthropogenic land-use is a critical factor^14^. When ecohydrological characteristics and plant physiological regulations in natural drylands are considered, adverse effects of warming and drying can be mitigated by reducing water losses from soils^15,16^. Therefore, the real contribution of land use and climate conditions to current and future changes in natural vegetation cover needs to be verified at the local- and regional-scale for drylands.

Land-use change has converted 50% of the world’s natural land cover over the last 300 years^17^. Drylands in the Neotropics, for instance, are old colonization frontiers that are mostly covered by second-growth vegetation^18^. Empirical data suggests that long-term land use has reduced the resilience of the remaining natural vegetation in semiarid regions, with the rainy season offering little in the way of a boost to rates of recovery^19^. Landscape structure also plays a major role in controlling and defining successional vegetation trajectories in dry tropical regions, as faster recovery occurs in sites surrounded by higher forest cover^20^. Therefore, in addition to desertification, second-growth vegetation can represent a degradation-driven alternative stable state in dry forest regions^21^. Forest degradation is defined as a state of anthropogenically-induced arrested succession, where ecological processes that underlie forest dynamics are disturbed or severely constrained^22^. Consequently, alternative stable states via forest-to-shrub conversions can be found^23^, and these phenomena can mask the real interaction between climate and natural vegetation cover in semi-arid regions.

The Caatinga region is one of the most populated and biodiverse seasonally dry tropical forests (SDTF) globally, which has undergone intense transformations (including desertification) over the past five centuries, increasing its vulnerability to climate change^24^. Historical records from the eighteenth and nineteenth centuries demonstrate that the Caatinga was characterized by extensive dry forests with high levels of biodiversity^25,26^. However, slash-and-burn agriculture, free-ranging livestock, exploitation of forest products, and cycles of intensive agriculture (e.g., cotton) have converted large tracts of old-growth forest into vegetation mosaics dominated by shrubby vegetation and regenerating forest stands, areas of bare soil, and agricultural fields and pastures, across extensive areas during the twentieth and twenty-first centuries^27^. Based on remote sensing data, some estimates suggest that the Caatinga has more than 60% natural vegetation cover^28,29^, but these estimates consider all shrubland as natural vegetation and do not consider historical forest degradation caused by forest-to-shrub conversions (Table 1). Thus, the potential ecosystem services provided by undisturbed natural vegetation may be underestimated in the Caatinga, since soil and vegetation carbon stocks drop drastically following land-use change, even from dry forest to shrubland, for instance^30^. Nevertheless, we recognize that distinguishing between degradation-induced forest-to-shrub conversion and undisturbed natural shrubland is hard since this is also a natural vegetation type throughout the region, and remains a challenge.

**Table 1:** Ground and aerial views of the land use and land cover (LULC) found in the Caatinga region.

View		LULC type	
Ground	Aerial	Present paper	MapBiomas
		Forest (*no change*)	Forest formation
		Shrub (*no change*)	Savanna formation
		Forest to shrub (*degradation–level 1*)	Savanna formation
		Forest/shurb to farming (*degradation–level 2*)	Pasture or agriculture
		Forest/shurb to non vegetation (*degradation–level 3*)	Other non vegetated areas

The LULC types used in this paper were classified in degradation levels (italics) and contrasted with LULC types derived from current satellite data (MapBiomas^29^).

In this context, paleoecological patterns (including faunal composition) can provide insights into the relationships between climate and natural vegetation types^31^. Due to past climate change, larger-sized mammals were narrowly distributed across scattered patches of suitable habitats in South America throughout the late Quaternary^32^. In the Holocene, the loss of larger-sized mammal lineages was related to the decrease of open vegetation areas and an increase in dry forests in South American semiarid regions^33^. On the other hand, the life-history traits of current endemic bird species associated with different vegetation types (forest or shrubland, for instance) support the occurrence of a natural gradient from forest to open vegetation composing the original cover in South American drylands^34^. However, some current species associated with open vegetation can increase their distribution because they benefit from anthropogenic disturbance, and, therefore, these contemporary changes hamper efforts to identify current patterns of natural vegetation distribution.

Here, we (1) estimate the potential distribution of natural Caatinga vegetation cover, considering climate and soil; (2) estimate land-cover change; and (3) identify the relative contribution of humans and climate as drivers of these land-cover changes. Three questions guided the study: (a) What would the expected distribution of dry forest and shrubland be in the absence of acute human disturbance? (b) How much of the natural distribution has been modified? (c) What are the relative contributions of human activities and climate conditions to vegetation change in distinct degradation transitions (forest to shrub, forest/shrub to farming, forest/shrub to non-vegetation) (Table 1)? To answer these questions, we used potential distribution models of strictly forest-dependent endemic birds as a proxy of forest habitats and potential distribution models of extinct larger-sized mammals for LGM, projected to current climate conditions, as a proxy of shrubland distribution across the region. Further, (1) these models were contrasted with the present-day vegetation across the entire region, and (2) the documented vegetation changes were contrasted with anthropogenic and climatic variables.

## Results

### Potential vegetation distribution

**Figure 1:**
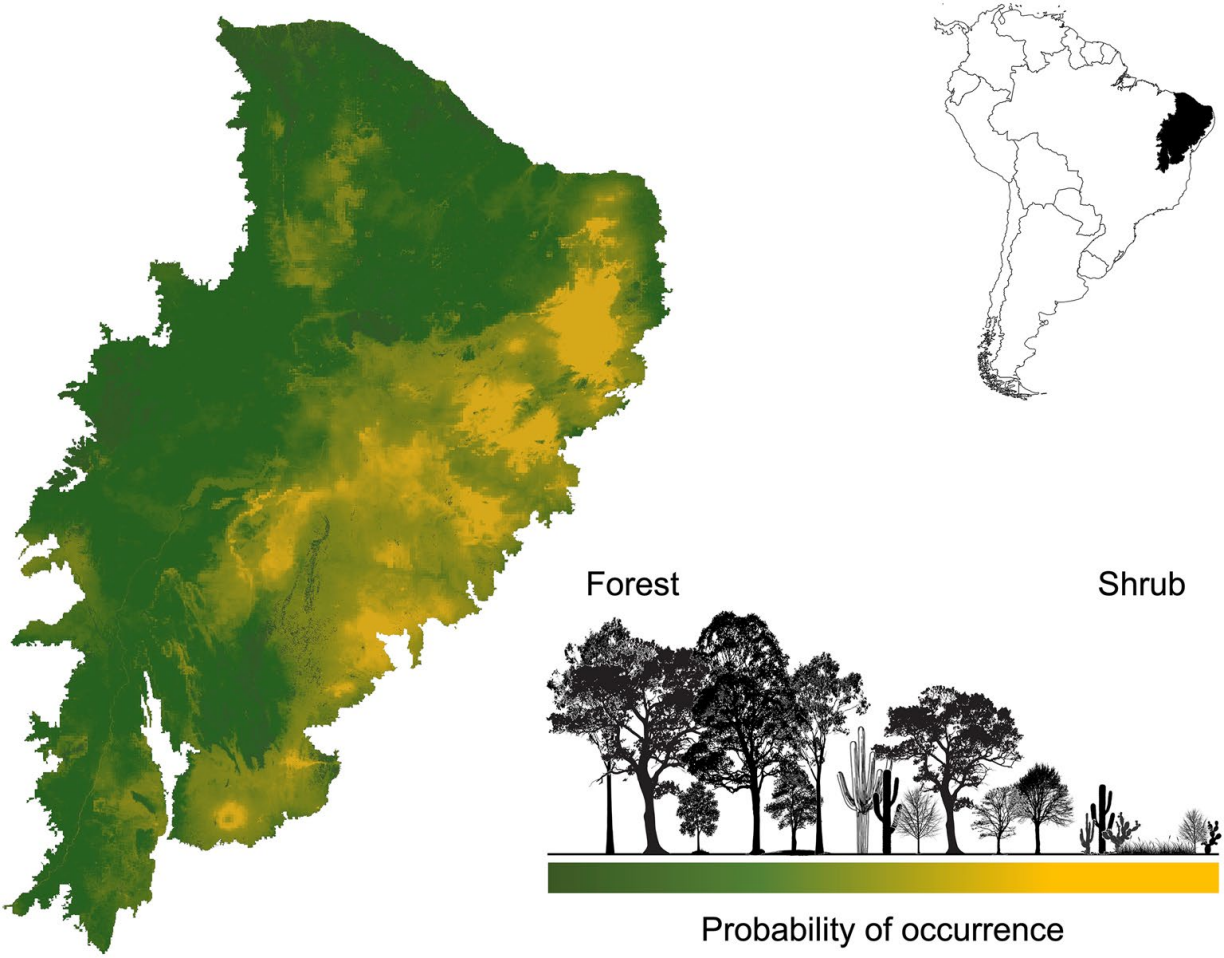
Potential distribution of forest and shrubland in the Caatinga for current climatic and soil conditions. The map was generated from the Maxent model results (see methods for details) using the software QGIS version 3.20.0-Odense (https://qgis.org/en/site/).

Our vegetation distribution models indicated a high probability of potential forest distribution stretching across the south, center, and west of the Caatinga region, and patches in the east. On the other hand, our models indicated a high probability of shrub habitats occurring in the Caatinga, particularly in the center and east of the region (Fig. 1, Supplementary Fig. S1). All models were influenced by environmental variables, with area under the curve (AUC) values greater than 0.8 for all models (i.e., a non-random distribution of vegetation types). Of the environmental variables we assessed, the mean temperature of wettest quarter (25.3%), temperature seasonality (16.9%), and annual precipitation (13.5%) were the most important, explaining 55.7% of forest distribution. While annual precipitation (57.3%) and annual mean temperature (10.7%) explained 68% of the distribution of shrub habitats (Supplementary Table S1).

### Change in vegetation cover

**Figure 2:**
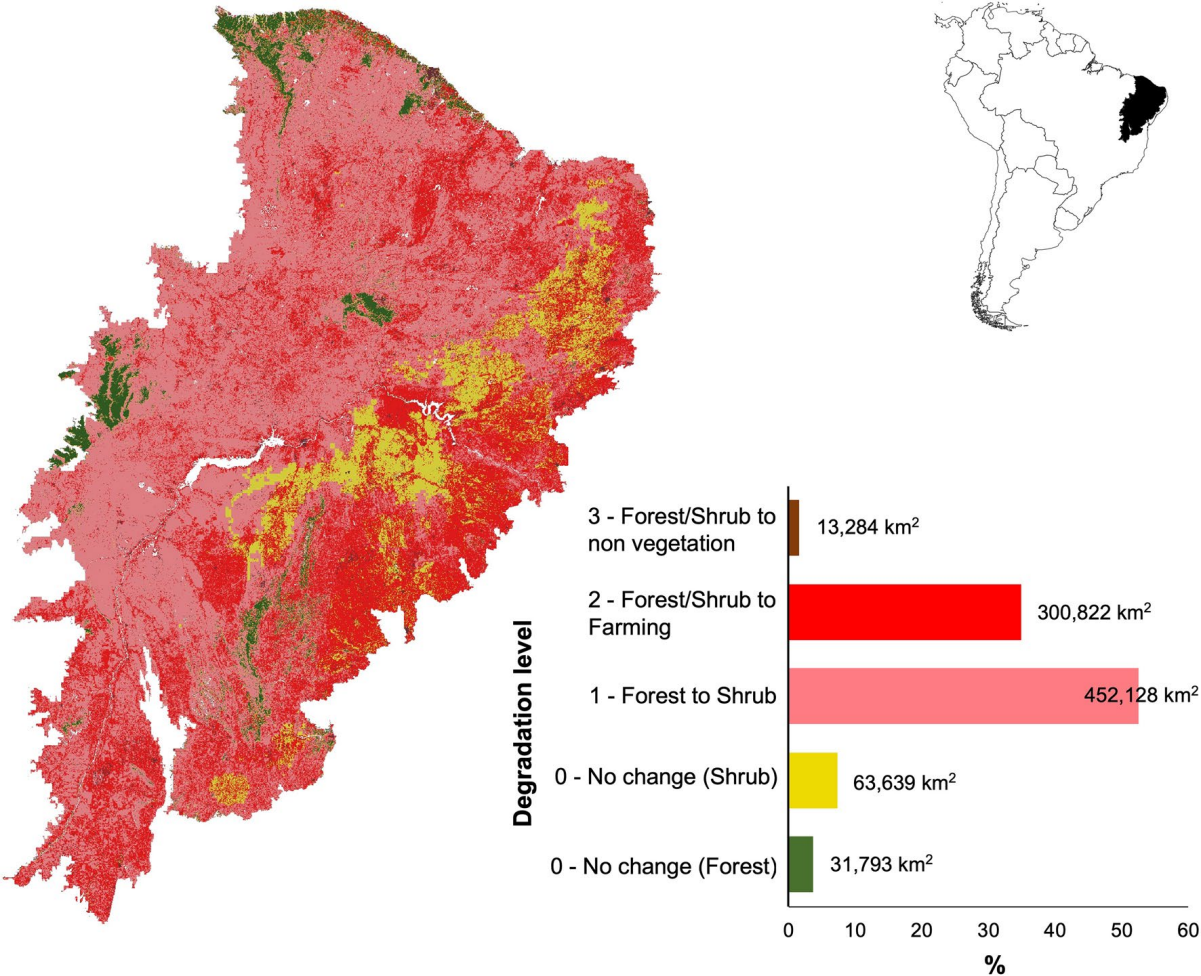
Distribution and amount of each forest degradation level in the Caatinga region. Forest degradation is defined as a state of anthropogenically induced arrested succession, where ecological processes that underlie forest dynamics are diminished or severely constrained^22^. The map was generated by comparing potential distribution and current land cover (see methods for details) using the software QGIS version 3.20.0-Odense (https://qgis.org/en/site/).

Considering the spatial limits of the Caatinga biota (i.e., 863,752 km^2^), we predicted a total potential area of 731,211 km^2^ for forest cover, and 132,195 km^2^ for shrub cover, which corresponds to approximately 84.6% and 15.3% of the entire region, respectively. However, our analysis of present-day cover indicated that only 31,793 km^2^ is covered by forest, 63,639 km^2^ by natural shrubland, and 766,235 km^2^ by degraded vegetation (452,128 km^2^ by forest to shrubland, 300,822 km^2^ by forest/shrubland to farming, and 13,284 km^2^ by non-vegetation cover) (Fig. 2). These scores indicated an extensive loss of forest, i.e., only 4.34% of their potential distributions remaining. On the other hand, shrubland has expanded by 390% by grouping undisturbed natural shrubland and forest to shrubland change (Fig. 2). Furthermore, 35% were modified to farming and 1.6% to non-vegetation cover; i.e. desertification areas.

### Drivers of vegetation cover change

**Figure 3:**
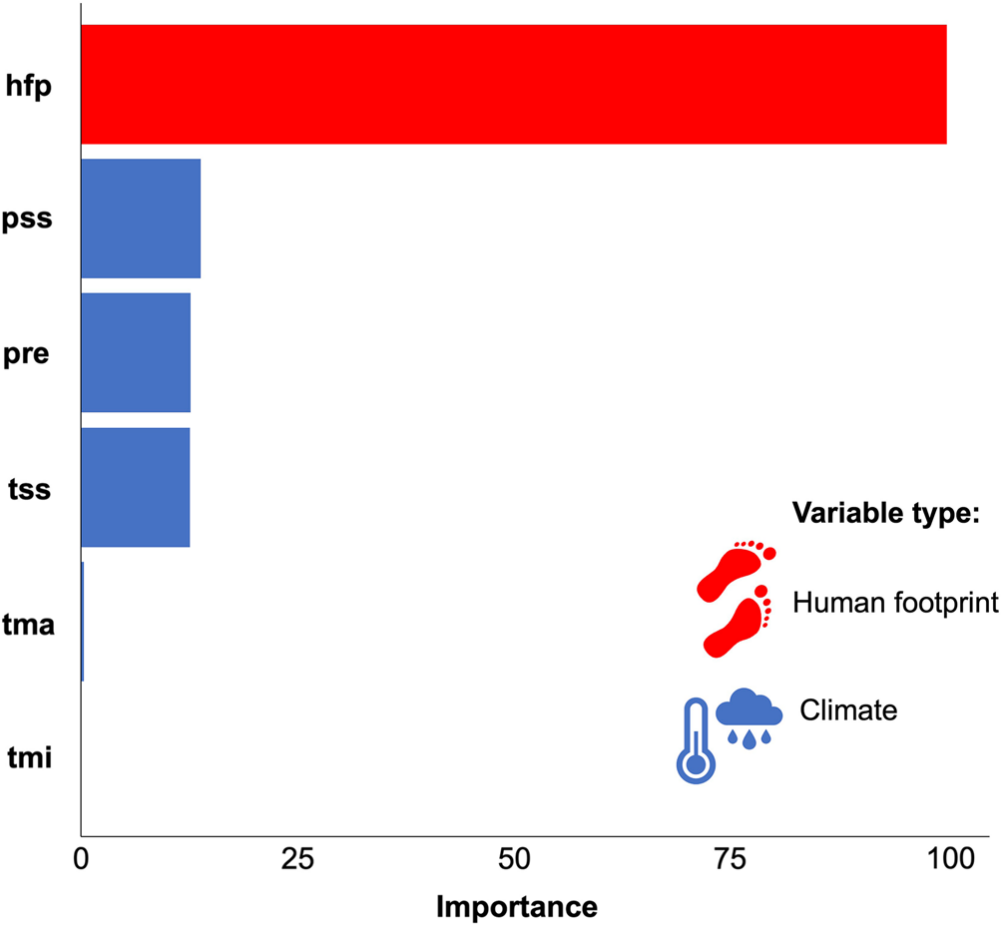
Importance values of human footprint (hfp) and climate variables on the forest degradation in the Caatinga region. Importance values were calculated based on a Random Forest model that explained 73% of the variation in the data. Climate variables were: precipitation seasonality (pss), annual precipitation (pre), temperature seasonality (tss), max temperature (tma), and min temperature (tmi).

Our random forest model predicted c. 73% (R^2^ = 0.73, MAE = 0.14, RMSE = 0.20) vegetation cover change. The human footprint was the variable with the highest relative importance (100%), consistently appearing in all models and the best-performing model. On the other hand, climatic variables (precipitation seasonality, annual precipitation, and temperature seasonality) contributed less than 15% each (Fig. 3). However, the highest degradation levels were weakly associated with areas where current temperature seasonality is higher, and annual precipitation and precipitation seasonality are lower (Supplementary Fig. S2).

## Discussion

Our results indicate that the Caatinga has climatic and soil conditions able to support more forest cover than is currently found throughout the region, regardless of the current occurrence of low-precipitation areas (Supplementary Fig. S3). However, our estimates indicate that only 11.04% of native vegetation cover persists across the whole region, with only 4.34% being forest cover. On the other hand, forest degradation is characterized by the dramatic expansion of shrubland (390%), farming, and non-vegetation cover due to changes in land-use, rather than climatic conditions. Climate conditions have been the major driver for forest and natural shrubland distribution in the Caatinga region, with areas climatically suitable for both vegetation types. However, the forest seems unable to resist the consequences of land-use changes, particularly in those areas where the temperature seasonality is high and annual precipitation is low. The patterns we have uncovered highlight the resistance the Caatinga has to different climatic conditions while reinforcing the prominence of land-use change as the main driver of the rapid decline in forest cover and the proliferation of reduced tree-cover vegetation types such as shrublands.

Although our approach was able to identify the potential forest distribution across areas with past forest occurrence, confirmed by historical records from the eighteenth and nineteenth centuries^25,26^, it is worth mentioning the potential limitations of the model sensitivity. Even using climate extremes and soil variables to predict potential vegetation distribution cover at a regional scale (Supplementary Table S2), local conditions may support particular vegetation types, which are not detected by regional-level assessments. However, our findings indicated that the forest and shrubland could potentially occur across a wide range of climate conditions, which naturally reduces the prominence of local conditions (particularly soil conditions) as the main driver of vegetation type. This perspective is supported by the present-day occurrence of Caatinga dry forest across a wide range of soil conditions, such as deep sandy soils to shallow and clay-based relatively rich soils^35^.

Globally, precipitation and water availability are the major drivers of vegetation distribution^36^ by controlling biological processes from plant physiology to ecosystem dynamics and structure^37^. In the Caatinga region, the precipitation regime is mainly associated with warm and humid air masses coming from the west (Continental Equatorial), southeast (Atlantic Tropical), and north-northeast (Atlantic Equatorial), as well as with the relief which contributes to the occurrence orographic rains^38^. The Caatinga biota dates back to the Miocene and since then it has experienced climate changes^39,40^. Moreover, the Caatinga dry forest stretches over a wide spectrum of soils and precipitation conditions, which results in gradients of water availability occurring from local to regional spatial scales. In addition to successive climate changes, the Caatinga biota has long been exposed to frequent droughts, commonly associated with El Niño Southern Oscillation events^41^. This evolutionary and ecological context has probably favored plant lineages able to cope with increased ecological filtering such as those with conservative resource-use strategies, high plasticity, and the ability to resprout as previously documented^42^. In fact, the resistance of the Caatinga to different climatic conditions and its ability to cope with climate oscillations through time has been considered a key asset in a global change scenario^43^. However, this apparent resistance to climate oscillations has not been sufficient to resist local human disturbances, resulting in the expansion of shrublands and deserts^44^. The Caatinga has been subjected to intense human disturbances since the arrival of Europeans in the sixteenth century^45^. We refer to an immense package of disturbances including slash-and-burn agriculture, extensive cattle raising, and the exploitation of forest products. Such disturbance is varied and intense including the collection of firewood, wood for charcoal production, timber, and fodder, plus the periodic cycles of commercial agriculture devoted to commodities such as cotton^46–48^.

Our findings confirm land-use change as the main driving force of forest loss and distinctive successional trajectories towards shrublands and anthropogenic-induced deserts. This trajectory affects ecosystem services provided by the natural vegetation cover, such as soil/vegetation carbon stocks, which drastically drop in the case of biomass, when forest, for example, is converted to shrublands^30,43,44^. Present-day satellite data indicates that aboveground forest biomass can reach 80–130 Mg/ha in the Caatinga, but such a high-biomass forest covers only about 7% of the region today, while 73% of its area support aboveground biomass < 40 Mg/ha^49^. Landscape-level data obtained through direct measurements have also found reduced aboveground forest biomass across successional mosaics resulting from slash-and-burn agriculture^27^, with several sites exhibiting low rates of biomass recovery (i.e. reduced resilience). We refer to secondary/regenerating forest stands (up to 70-yr old) limited to 50 Mg/ha on average^49,50^. In fact, dry forest regeneration and dynamics are influenced by local land use and landscape context, where long-term land use affects the potential for recovery, particularly when soils are degraded^51^, regeneration mechanisms (e.g. seeds and sprouts) are compromised, and sites are located far from old-growth forest patches^19,20^. In other words, forest persistence has already been compromised across the majority of the Caatinga region, because more than 90% of the potential forest distribution has already been modified, as a result of disruptions in forest regeneration mechanisms at the regional scale. Therefore, the land-use change has exceeded ecological thresholds (i.e., tipping points) for the persistence of forests. This scenario demands active forest restoration initiatives across the entire region if society is to have any chance of protecting the irreplaceable biodiversity and ecosystem services of the Caatinga.

Considering the socioecological context of the Caatinga, with the prevalence of subsistence farming, we argue that this transition of dry forests naturally dominated by single-stem tree species towards deserts (i.e. bare soils) or vegetation type dominated by multi-stemmed shrubs (here defined as shrubland) is a response by the forest ecosystems to a continuous exposition to conversion into pastures^17,52^, slash-and-burn agriculture^53^, shifting cultivation^27,54^, coppicing^23^, and browsing by livestock^48,55^, as well as the exploitation of forest products. Such an alternative successional trajectory is not exclusively related to tree species replacement by shrub species, but also by the predominance of tree species occurring as multi-stem shrub-sized individuals, as is already occurring with almost all dominant tree species in human-modified Caatinga landscapes^56–58^. Such forest transition towards shrublands as an alternative successional trajectory has been previously proposed for other dry forests exposed to intensive land use^59^. However, to what extent regeneration favoring shrubs rather than trees (one of the stages towards human-induced desertification in the Caatinga) meets the concept of alternative stable states, remains to be investigated.

In synthesis, natural cover in the Caatinga has been replaced by deserts^44^ and shrublands due to historical and current anthropogenic land use. Land-use intensification is already causing tree cover and aboveground biomass decline, not only due to deforestation but also due to changes in the structural status of the remaining vegetation, such as the spread of shrublands, at the expense of forests. This transition toward treeless land cover reduces ecosystem services, as is already occurring in tropical dry forests around the world (i.e. forest degradation), affecting billions of people who directly depend on forest products and their regulating services^7,12,59^. Although much attention has been given to the occurrence of potential precipitation thresholds which, if exceeded, may permanently reduce tree cover and biomass, comparatively little attention has been paid to land-use intensification, which is already causing fast declines in tree cover and biomass. However, climate change may be a synergistic force that will intensify (where precipitation is reduced and drought events are more frequent) or retard (where precipitation increases) regeneration dynamics representing alternative trajectories in human-modified landscapes^15^. In this context, it might be misleading to adopt current patterns of vegetation distribution across human-modified landscapes as a reliable indicator to infer vegetation sensitivity to climate change. Finally, we propose that landscapes composed of a combination of forests and crop and/or cattle farming may allow high food productivity, biodiversity, and ecosystem services, even during extreme drought events^60,61^. However, dry forest restoration and the adoption of better practices to prevent further degradation are urgently needed to help the recovery of ecosystem productivity and resilience for the sake of global sustainability.

## Methods

### Study area

The Caatinga region in northeast Brazil is one of the world’s largest SDTFs^62^, which originally covered almost one million km^2^^63^. This biota is restricted to the Brazilian territory, and it covers crystalline basement surfaces and sand sedimentary basins supporting flattened surfaces cut by narrow valleys, residual hills, and high-altitude plateaus (Supplementary Fig. S4). Soil types are diverse, ranging from shallow, rocky, and quite fertile to deep, sandy, and unfertile. The climate is classified as BSh (hot semi-arid) and As (with dry summer) across most of the region^64^. Most of the rainfall is concentrated in three consecutive months, but the region experiences spatial and annual variations. Due to the great inter-annual variability of precipitation, droughts can endure for years^38^.

At the regional level, the Caatinga dry forest was originally a vegetation mosaic dominated by dry forest stands of varying structure (high- to low-statured forests)^65^, which also supported enclaves of both semideciduous rainforest and Cerrado (i.e. Brazilian savanna), particularly due to the occurrence of patchy orographic rain. The Caatinga dry forest is highly diverse, with levels of species endemism ranging from 5 to 25%^65^. This unique biota has been intensively modified since the arrival of Europeans in the sixteenth century by a combination of (1) slash-and-burn agriculture, (2) cycles of intensive agriculture devoted to commodities across particular sites, (3) free-ranging livestock (principally goats and cattle), and (4) exploitation of forest products, such as firewood for domestic use and for charcoal production, fodder, and poles for farming facilities^46^. Rural smallholdings are prevalent throughout the region, with residents’ livelihoods depending on natural resources and ecosystem services such as nutrient provision for subsistence agriculture.

To address our questions, three complementary and integrative steps were performed as described below.

### Potential distribution of forest and shrub vegetation

The potential forest and shrub distribution at the biota scale under current climatic conditions was estimated using (1) taxa associated with forest habitats under present climate conditions, and (2) vertebrate taxa associated with open vegetation during the Last Glacial Maximum (LGM; 21 ka BP). More precisely, we used records of strictly forest-dependent endemic bird species as a proxy, since these species do not benefit from anthropogenic changes in the landscape. In the Caatinga, 31% of bird species are forest-dependent and such habitat filtering reduces the influences of spatial dynamics associated with the conservation status of the region^34^. Our database consisted of occurrence records of six endemic bird species: *Conopophaga cearae, Hylopezus ochroleucus, Xiphocolaptes falcirostris, Lepidocolaptes wagleri, Phylloscartes roquettei, Arremon franciscanus*. Species’ occurrences were retrieved from the avifauna literature and the following online databases: Global Biodiversity Information Facility (Gbif—https://www.gbif.org), Species link (http://www.splink.org.br), and Wikiaves (https://www.wikiaves.com.br).

We did not use present-day endemic species to model open vegetation cover because these species can benefit from anthropogenic processes that reduce forest cover^34^. Therefore, we used extinct megafauna occurrences in the Caatinga to model potential shrub-dominated environments. Occurrences of megafauna taxa that lived at the end of the Pleistocene were collected from papers on animal paleoecology. The interpretations of paleoecology were based on dental morphology and studies involving carbon isotopes. Taxa were selected based on literature focusing on grazing behavior, C4 plant-based diets (grasses), and occurrence in open areas (Supplementary Table S3). Our database consisted of occurrence records of eight species: *Equus neogaeus, Glossotherium sp., Glyptodon clavipes, Glyptotherium cylindricum, Pampatherium humboldtii, Panochthus greslebini, Panochthus jaguaribensis,* and *Xenorhinotherium bahiense*. The occurrences of the species were collected from bibliographic surveys of fossil findings in the Caatinga, paleontology collections (Department of Geology of the Federal University of Pernambuco, Vertebrate and Paleontology Laboratory of the Federal University of Paraíba), Federal University of Bahia (from Prof. Dr. Mario Dantas), and Paleobiology networks (https://www.paleobiodb.org).

Distribution modeling was carried out using 393 geographic points in the Caatinga region, 196 from forest cover indicators under current climatic and soil conditions, and 197 from shrub environment indicators under LGM (21 ka BP) climatic conditions. We used 19 climatic variables obtained from Worldclim (http://www.worldclim.org) and PaleoClim (http://www.paleoclim.org). The climatic variables used to model forest cover, obtained through present-day forest bird occurrence, were selected after an autocorrelation analysis of the current climatic conditions in the Caatinga region. Variables used for shrub environments were selected after an autocorrelation analysis of the LGM climatic conditions (Supplementary Table S2). We included in the models the variables with a correlation < 0.8 to avoid redundancy of climatic variables. We used Pearson’s correlation, calculated using the *Vegan* package for R (version 3.5.3). Physical soil properties (Clay and Sand content in g/kg) also were used as variables to model current forest cover. These variables were obtained from digital soil mapping (SoilGrids) based on a global compilation of soil profile data (WoSIS) and environmental layers (http://www.soilgrids.org). Further information about the SoilGrids and WoSIS projects is available on http://isric.org.

The distribution models were generated with the Maximum Entropy (Maxent) algorithm^66^, which models a probability distribution where each grid cell has predicted the suitability of conditions, from a set of environmental variables and georeferenced occurrence records. The highest value of area under the curve (AUC) was used to select the best models. An AUC ≥ 0.75 indicated that the modeled distribution was not random but influenced by environmental variables. We use Receiver Operator Characteristic statistics (ROC) to assess model accuracy with 10 repetitions of 10,000 interactions, with 10% of replicas being randomly selected as test data and the remaining 90% used for model training in each replica.

To map the potential distribution of natural vegetation cover, we summed the raster file of the current potential distribution of forest with the reprojected raster file of the current potential distribution of shrubs, derived from the LGM model. For that, we reproject the LGM model to the current climate scenario using Maxent and used the raster calculator to multiply the likelihood of shrub occurrence by − 1. So, we summed the positive probability (0 to 1) for the potential distribution of forest with the negative probability (0 to − 1) that represents the potential distribution of shrub environments.

### Estimating vegetation cover change

To assess regional changes in vegetation cover, the potential distribution of forest and shrub vegetation for current conditions was compared with present-day vegetation cover, assessed by MapBiomas^29^ for the year 2021. We use current forest-bird occurrence data to calibrate the threshold expected of the probability distribution of forest, transforming the raster file of the potential distribution of natural vegetation covers into a binary map (forest vs shrub). This binary map was compared with MabBiomas land-use and land-cover type, and the difference was categorized in forest degradation levels (0—no change, when forest or shrub remained in Forest and Savanna Formation by MapBiomas, respectively; 1—forest to shrub, when Savanna Formation occur where the forest was predicted; 2—forest/shrub to farming, when Pasture or Agriculture occur where forest or shrub was predicted; 3—forest/shrub to non-vegetation, when Non-Vegetated Areas occur where forest or shrub was predicted (Table 1).

### Drivers of vegetation cover change

**Figure 4:**
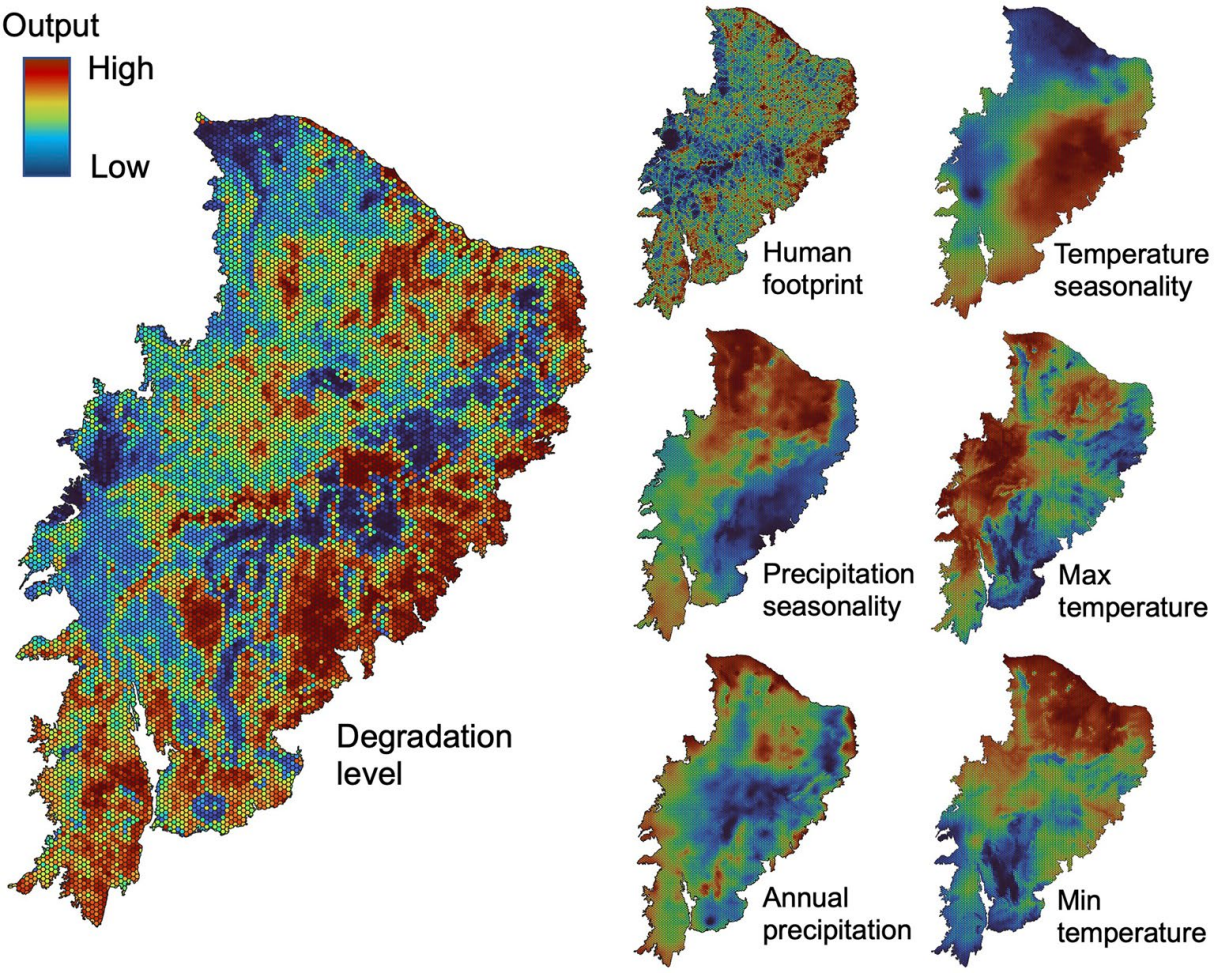
Distribution of the degradation levels (as a response variable) and human pressure and climate variables (as predictor variables) in the Caatinga region. The relationship between the predictor variables and the response variable was evaluated using a Random Forest predictive model (see results and Fig. 3). The maps were generated using the software QGIS version 3.20.0-Odense (https://qgis.org/en/site/).

To identify the relative contribution of humans and climate as drivers of the land-cover changes, we used a hexagonal grid covering the entire Caatinga. We use 12,976 hexagons with a side of 5km (area 64.95 km^2^), and statistical zonal to obtain information from degradation level as response variable and human footprint and climate as predictor variables. The degradation level raster was obtained from the previous step, where each pixel (30 × 30 m) has a value between 0 and 3 (0—no change, 1—forest to shrub, 2—forest/shrub to farming, 3—forest/shrub to non-vegetation). The Human Footprint (hfp) was used as a human pressure variable and was obtained from a global map terrestrial Human Footprint^67^ (1 km^2^ resolution). The Human Footprint map indicates human pressure weighted within that range according to estimates of their relative levels of human pressure for the extent of built environments, population density, electricity infrastructure, crop lands, pasture lands, roads, railways, and navigable waterways^67^. Climate variables used as predictors were precipitation seasonality (pss), annual precipitation (pre), temperature seasonality (tss), max temperature (tma), and min temperature (tmi) (1 km^2^ resolution). These climate data were obtained from Worldclim^68^. We extracted the mean value of each variable from each hexagon (Fig. 4).

To evaluate the relationship between the predictor variables and the response variable, we used the Random Forest (RF) predictive analysis (Predictive Model with Machine Learning)^69^. In general, the RF algorithm is robust and one of the most used in predictive model analysis. It has few assumptions and data pre-processing and generation of predictive models with high performance, designed to avoid overfitting^69^. Through decision tree algorithms, RF produces classifications or regression models, as in the case evaluated here. Tuning of the model parameters was carried out to increase the performance of the model. In our RF analysis, hfp, pss, pre, tss, tma, and tmi are used as predictor variables, and degradation level (0 to 3) as the response variable. Analyses were performed using the caret package in R^70^.

To evaluate the performance of the models, RMSE (Root Mean Square Error), R^2^, and MAE (mean absolute error) were used. We selected the best model using the bestTune and finalModel functions from the caret package. In the final model, the selected variables are organized in order of importance. The importance of the variable is a value that measures the number of times the performance of the model increased when a specific predictor was included in the model. Therefore, the importance of the variable is closely related to the performance of the model and the relative values are scaled between 0 and 100^70^.

### Supplementary Information

Supplementary Information.

## Data Availability

The datasets used in this study are available from the corresponding author upon reasonable request.
