# Supplementary material for: Human disturbance is the major driver of vegetation changes in the Caatinga dry forest region
Source: Sci Rep. 2023 Oct 27;13:18440. doi: 10.1038/s41598-023-45571-9 (PMC10611708; doi:10.1038/s41598-023-45571-9)
Supplement: Supplementary file 1 — Supplementary Information. [file 41598_2023_45571_MOESM1_ESM.pdf]

Supplementary Information for

Human disturbance is the major driver of vegetation changes in the Caatinga dry forest region, and climate conditions can exacerbate them

Helder F. P. Araujo*, Nathália F. Canassa, Célia C. C. Machado, Marcelo Tabarelli

* Corresponding author: Helder F. P. Araujo
Email: helder@cca.ufpb.br

This PDF file includes:

Figures S1 to S4
Tables S1 to S3

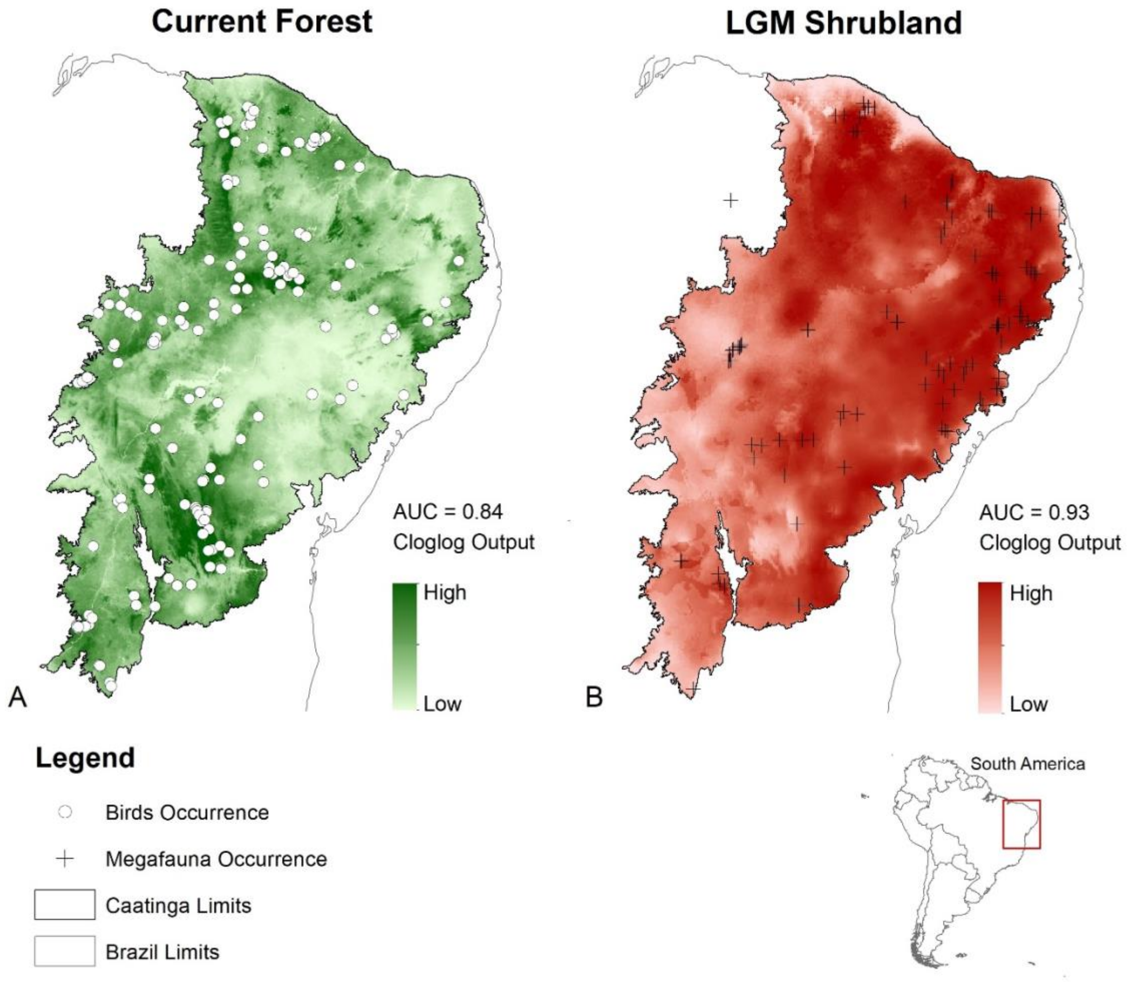

Supplementary Figure S1. Potential distribution of forest for the current period (A) based on forest-dependent bird species, and shrubland for Last Glacial Maximum (LGM) (B) based on megafauna occurrence. The colors in the cloglog output for each map represent the probabilities of the potential distributions. The maps were generated from the Maxent model results (see methods for details) using the software QGIS version 3.20.0-Odense (https://qgis.org/en/site/).

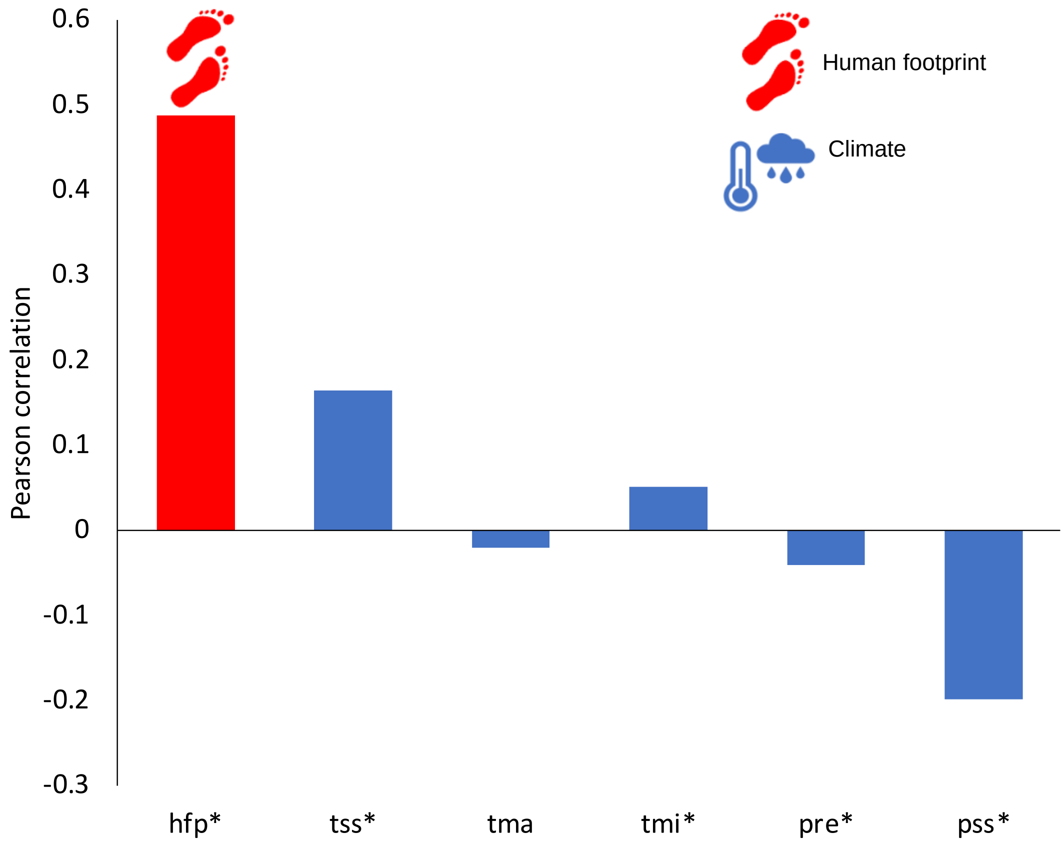

Supplementary Figure S2. Pearson correlation values demonstrating possible associations between forest degradation and potential drivers (human footprint (hfp) and climate variables in the Caatinga region. Climate variables refer to temperature seasonality (tss), max temperature (tma), min temperature (tmi), annual precipitation (pre), and precipitation seasonality (pss). (*) means significant association ($p<0.001$).

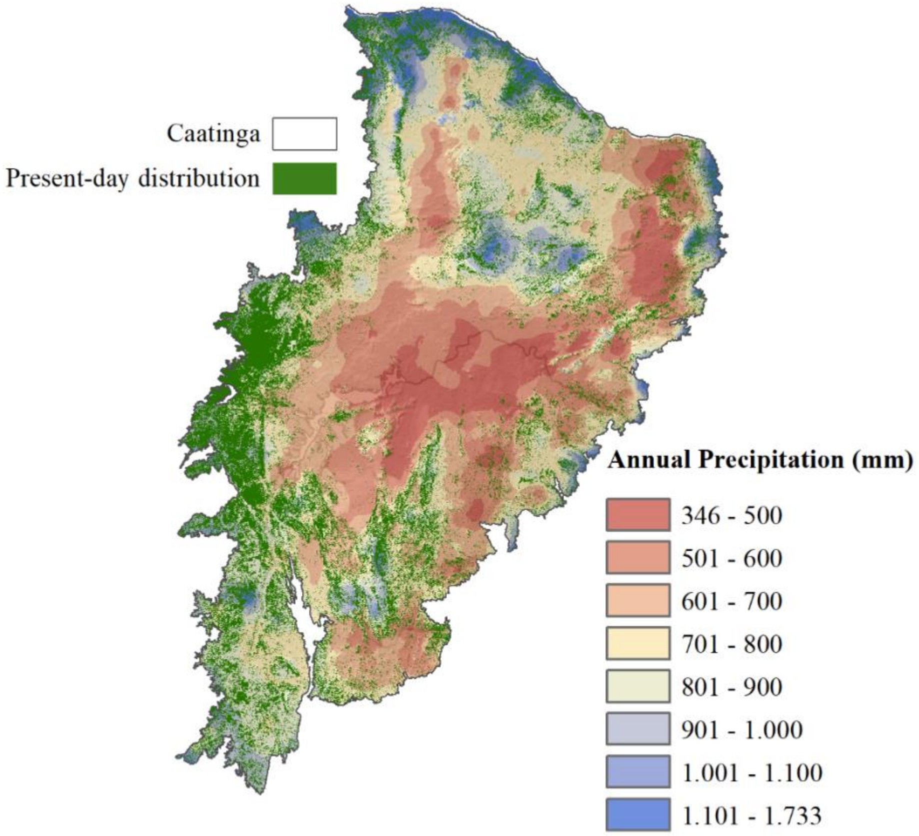

Supplementary Figure S3. Present-day distribution of forest and woodland (green), and average annual precipitation distribution across the Caatinga region. The map was generated using the software QGIS version 3.20.0-Odense (https://qgis.org/en/site/).

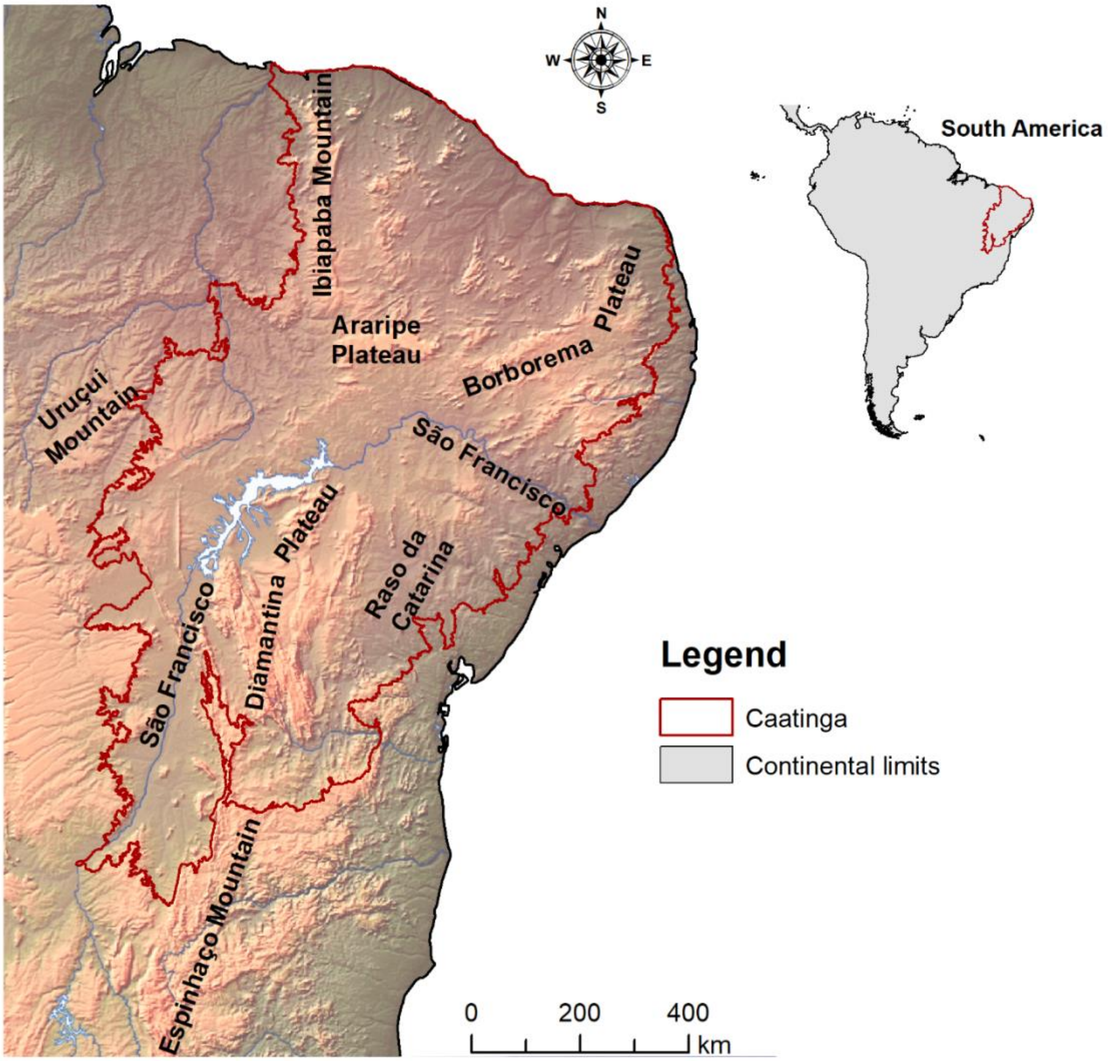

Supplementary Figure S4. The Brazilian dryland popularly and scientifically referred as Caatinga, the South American largest and more biodiverse tropical dry forest. It is a region with various landforms like flattened surfaces cut by narrow valleys, residual hills, high-altitude plateaus, and mountains that influence local precipitation distribution. The map was generated using the software QGIS version 3.20.0-Odense (https://qgis.org/en/site/).

Supplementary Table S1. Contribution from the e environmental variables for forest distribution models in the current period and shrub habitats during the Last Glacial Maximum (LGM).

Variables	Time	Cover	Contribution (%)
Annual Mean Temperature	Current	Forest	-
	LGM	Shrub	10.7
Mean Diurnal Range	Current	Forest	5.4
	LGM	Shrub	7
Isothermality	Current	Forest	2.1
	LGM	Shrub	1.4
Temperature Seasonality	Current	Forest	16.9
	LGM	Shrub	-
Temperature Annual Range	Current	Forest	-
	LGM	Shrub	7
Mean Temperature of Wettest Quarter	Current	Forest	25.3
	LGM	Shrub	-
Mean Temperature of Driest Quarter	Current	Forest	10.9
	LGM	Shrub	-
Annual Precipitation	Current	Forest	13.5
	LGM	Shrub	57.3
Precipitation of Wettest Month	Current	Forest	1.8
	LGM	Shrub	-
Precipitation of Driest Month	Current	Forest	5
	LGM	Shrub	-
Precipitation Seasonality	Current	Forest	6.9
	LGM	Shrub	0.1
Precipitation of Driest Quarter	Current	Forest	-
	LGM	Shrub	8
Precipitation of Warmest Quarter	Current	Forest	3.1
	LGM	Shrub	8.6
Precipitation of Coldest Quarter	Current	Forest	3.1
	LGM	Shrub	-
Soil (clay content)	Current	Forest	1.8
Soil (sand content)	Current	Forest	4.2

Supplementary Table S2. Environmental variables adopted for modeling potential distribution. Only uncorrelated variables were adopted. The current period was used to represent climate conditions used in the forest models. The Last Glacial Maximum (LGM) period was used to represent the climate conditions used in the open vegetation. The potential distribution from LGM was re-project to current for comparison. The “x” indicates which environmental variables were adopted in each period.

**Description**	**Current (1970-2000)**	**LGM (21k years BP)**
Annual Mean Temperature		x
Mean Diurnal Range		x
Isothermality		x
Temperature Seasonality	x	
Max Temperature of Warmest Month	x	
Min Temperature of Coldest Month		
Temperature Annual Range	x	x
Mean Temperature of Wettest Quarter	x	
Mean Temperature of Driest Quarter		
Mean Temperature of Warmest Quarter		
Mean Temperature of Coldest Quarter		
Annual Precipitation	x	x
Precipitation of Wettest Month		
Precipitation of Driest Month		
Precipitation Seasonality	x	x
Precipitation of Wettest Quarter		
Precipitation of Driest Quarter		x
Precipitation of Warmest Quarter	x	x
Precipitation of Coldest Quarter	x	

Supplementary Table S3. Extinct megafauna species in the Caatinga used to model the potential distribution of open vegetation. Palaeoecological information adopted as proxies for open environments, which were associated with diet, grazing behavior, and habitat use (dental morphology, carbon isotopes, grazing behavior, diet of C4 plants (grass), and occurrence in open areas).

**Species**	**Paleoecology proxy**	**Source***
Equus neogaeus	Dental morphology and diet of C4 plants	1
Glossotherium sp.	The lips coupled with the tongue were used to pull out grass and herbaceous plants	2
Glyptodon clavipes	Habitat use like savannas and grazing behavior	3, 4
Glyptotherium cylindricum	Habitat use like savannas and grazing behavior	4
Pampatherium humboldtii	Dental morphology, carbon isotopes, and diet of C4 plants	5
Panochthus greslebini	Habitat use like savannas and grazing behavior	3, 4, 6
Panochthus jaguaribensis	Habitat use like savannas and grazing behavior	3, 4, 6
Xenorhinotherium bahiense	Scratch in dental morphology	7

*Source:

1. M. T. Alberdi, C. Cartelle, J. L. Prado, El registro Pleistoceno de Equus (Amerhippus) e Hippidion (Mammalia, Perissodactyla) de Brasil. Consideraciones paleoecológicas y biogeográficas. Ameghiniana 40, 173–196 (2003).
2. M. S. Bargo, N. Toledo, S. F. Vizcaíno, Muzzle of South American Pleistocene ground sloths (Xenarthra, Tardigrada). J. Morphol. 267, 248–263 (2006).
3. H. I. Araújo-Junior, K. O. Porpino, Assembleias fossilíferas de mamíferos do Quaternário do Estado do Rio Grande do Norte, Nordeste do Brasil: diversidade e aspectos tafonômicos e paleoecológicos. Pesqui. em Geociências 38, 67–83 (2011).
4. M. C. T. Xavier, M. A. T. Dantas, C. C. Silva-Santana, Megafauna Pleistocênica da microrregião de Senhor do Bonfim, Bahia. Estud. Geol. 28, 19–31 (2018).
5. M. A. T. Dantas, et al., Identificação taxonômica dos fósseis de mamíferos da caverna Toca Fria e Jatobá, Iuiú, Bahia: Inferências paleoecológicas e temporais. An. do 32° Congr. Bras. Espeleol. 2113, 433–438 (2013).
6. J. de A. da Silva, L. A. Leal, A. Cherkinsky, M. A. T. Dantas, Late Pleistocene meso-megamammals from Anagé, Bahia, Brazil: Taxonomy and isotopic paleoecology (δ13C). J. South Am. Earth Sci. 96, 102362 (2019).
7. K. de O. Nascimento, "Paleoecologia alimentar de Macrauchenia patachonica e Xenorhinotherium bahiense (Macraucheniidae: Litopterna: Mammalia) e o reconhecimento de seus nichos ecológicos," Univesidade Federal do Estado do Rio de Janeiro. (2019).
